# Supplementary figures and images for: Shape-to-graph mapping method for efficient characterization and classification of complex geometries in biological images
Source: PLoS Comput Biol. 2020 Sep 3;16(9):e1007758. doi: 10.1371/journal.pcbi.1007758 (PMC7494120; doi:10.1371/journal.pcbi.1007758)

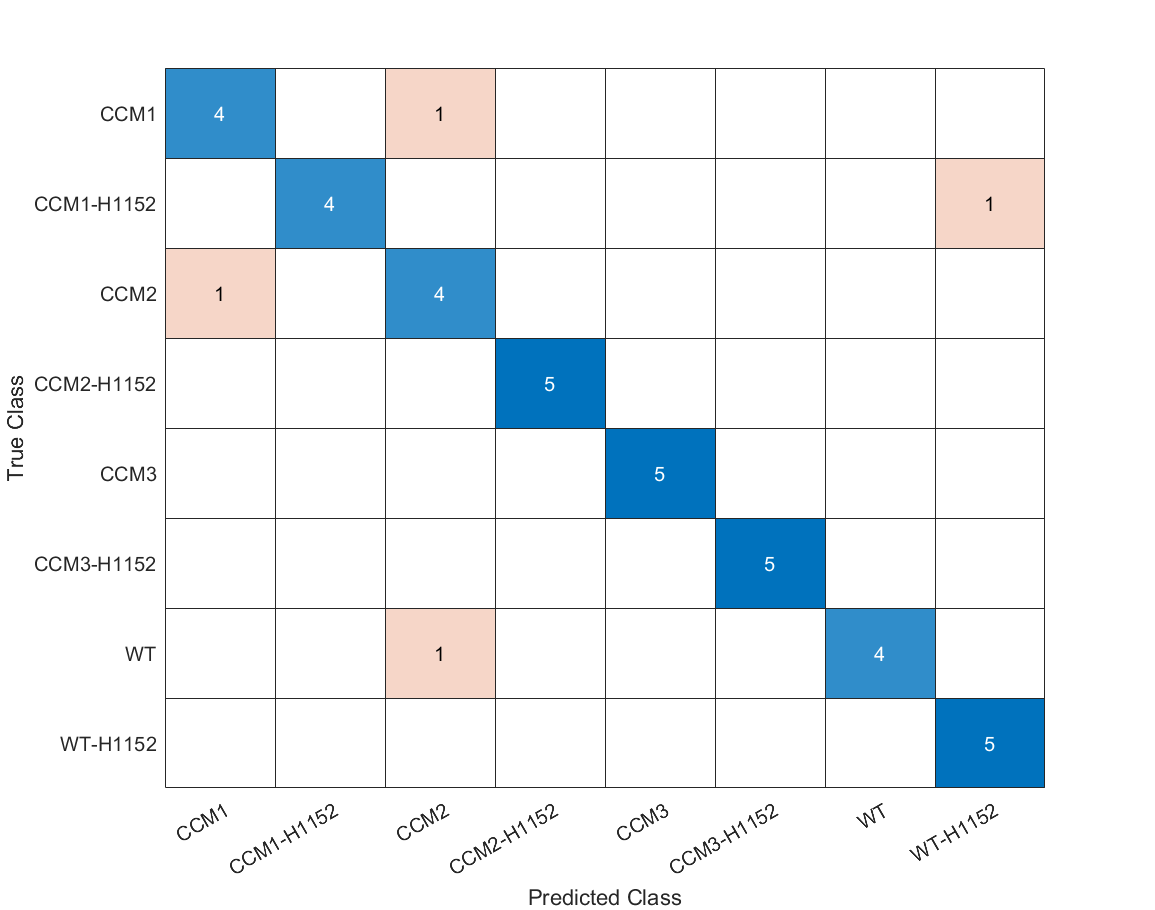

Supplement: S1 Fig — The results of 3-Nearest Neighbor classification on the CCM dataset using the shape-to-graph features. With 12 clusters, a 90% classification accuracy was achieved. Misclassifications occurred between the similar CCM1H1152 and WTH1152 structures, along with the CCM1 and CCM2 and WT and CCM2 structures, which have relatively few boundaries in each image. (TIF) [file pcbi.1007758.s001.tif]

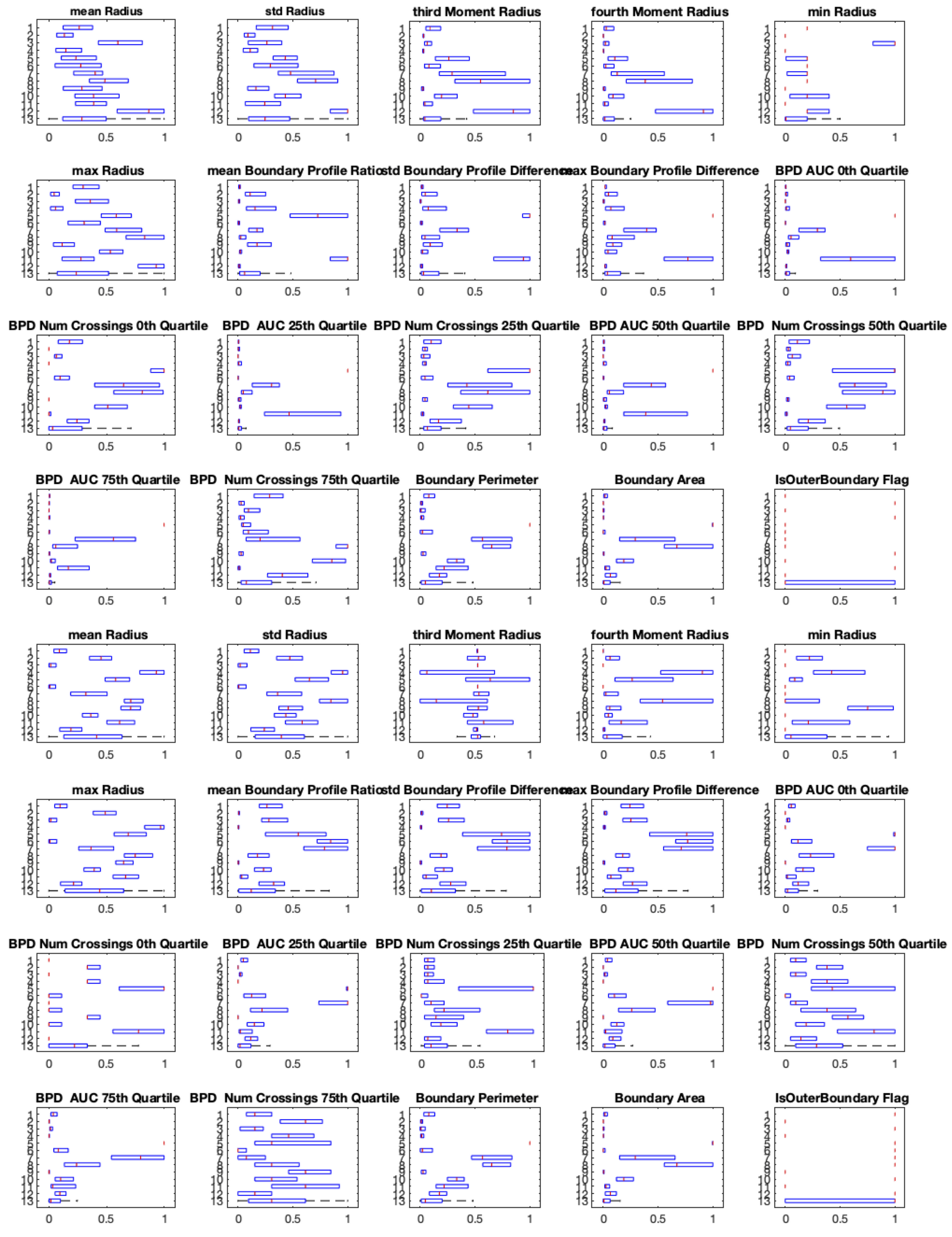

Supplement: S2 Fig — The distribution of each of the boundary metrics within a boundary type can be used to determine the characteristics of given boundary. Each boundary type accounts for information in the in-graph and out-graph surrounding the boundary. This means, for example, holes of similar size surrounded cell structure of different thickness may result in different boundary types. (TIF) [file pcbi.1007758.s002.tif]

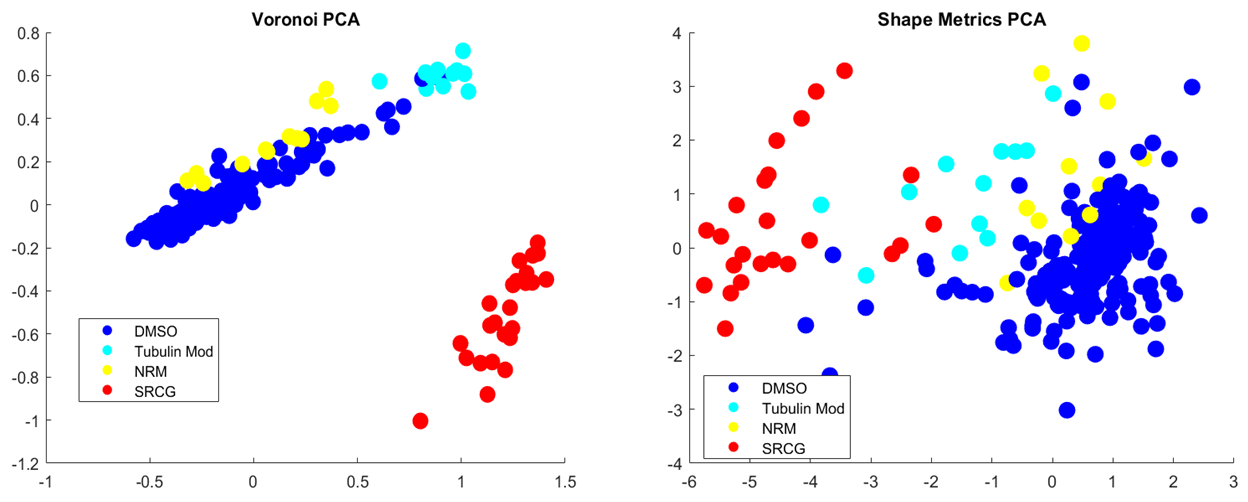

Supplement: S3 Fig — The first 2 principal components of the U2OS dataset with our new shape-to-graph metrics and the metrics in the original analysis. The metrics derived from our method creates tighter distributions with fewer overlaps between the chemical classes. (TIF) [file pcbi.1007758.s003.tif]

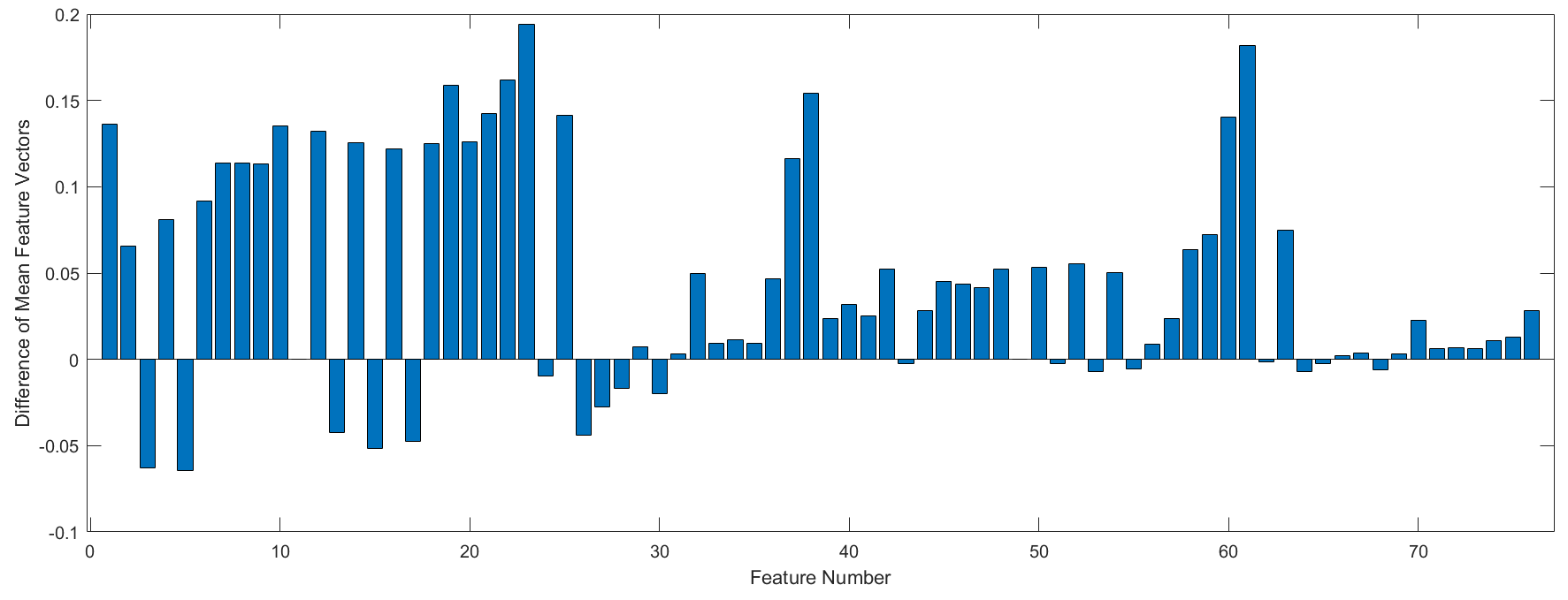

Supplement: S4 Fig — The difference of the average feature vectors between the control wells and the modulator of neuronal receptor wells can be used to see which features distinguish the classes. See S2 Table for descriptions of these features. Generally, features corresponding to the background radius, features 20–25 and 61–65, tend to be much larger in the modulators of neuronal receptors relative to control, indicating that the cells in this image are in a much less dense environment. (TIF) [file pcbi.1007758.s004.tif]

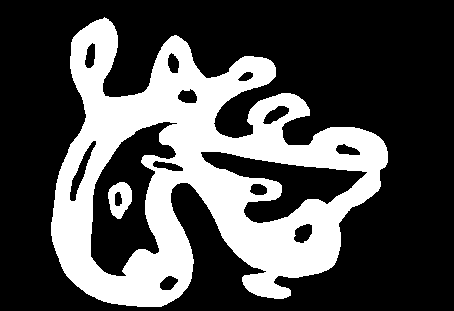

Supplement: S1 File — (ZIP) [file pcbi.1007758.s007.zip › SCRIPTs_and_GUIs/IMAGES for ShapeToGraph GUI/Example_2.tif]

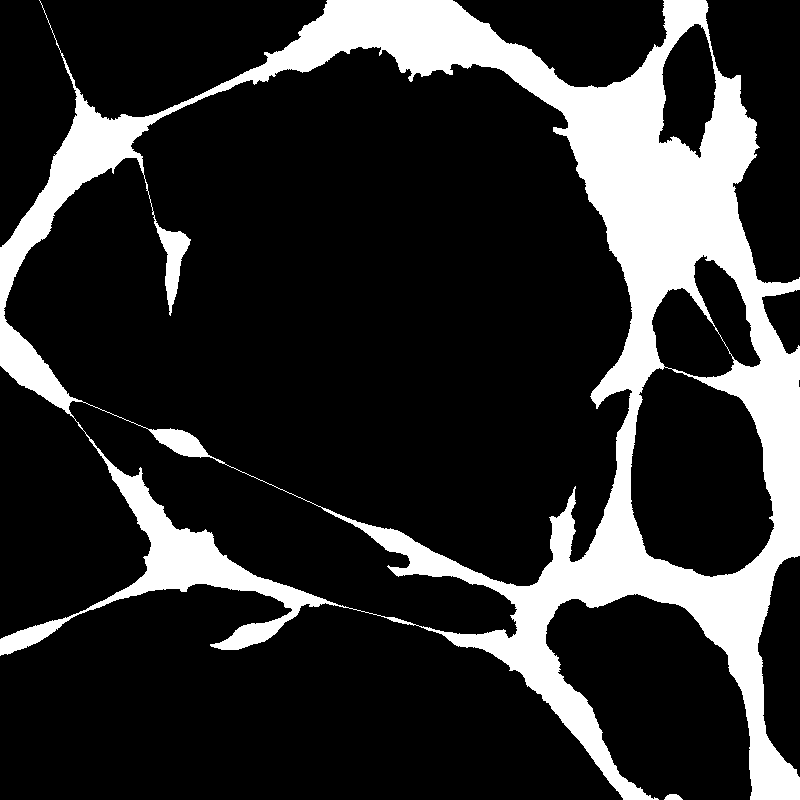

Supplement: S1 File — (ZIP) [file pcbi.1007758.s007.zip › SCRIPTs_and_GUIs/IMAGES for ShapeToGraph GUI/Example_3.tif]

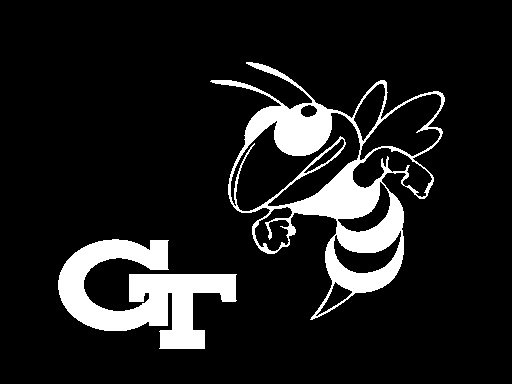

Supplement: S1 File — (ZIP) [file pcbi.1007758.s007.zip › SCRIPTs_and_GUIs/IMAGES for ShapeToGraph GUI/Example_1.tif]

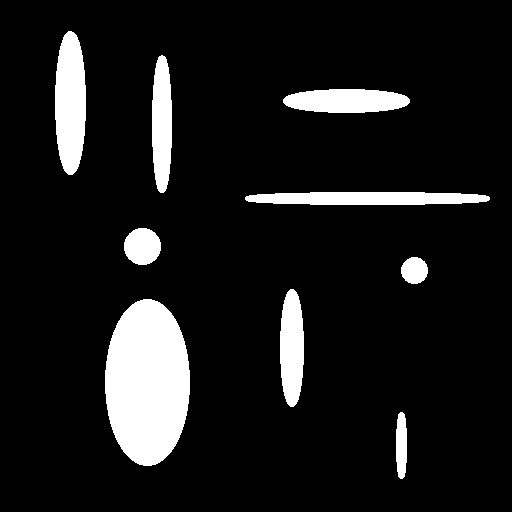

Supplement: S1 File — (ZIP) [file pcbi.1007758.s007.zip › SCRIPTs_and_GUIs/IMAGES for PCA GUI/ellipses7.png]

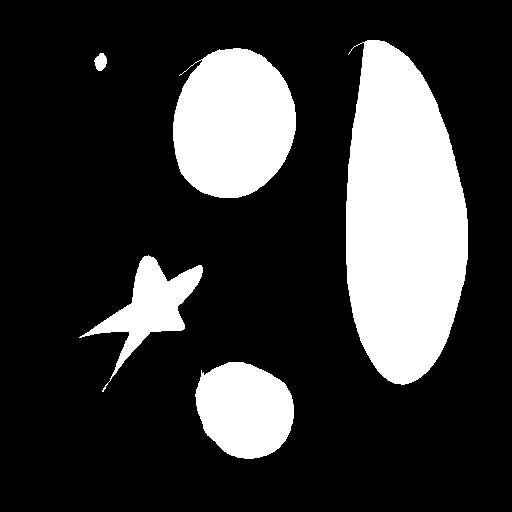

Supplement: S1 File — (ZIP) [file pcbi.1007758.s007.zip › SCRIPTs_and_GUIs/IMAGES for PCA GUI/manual11.png]

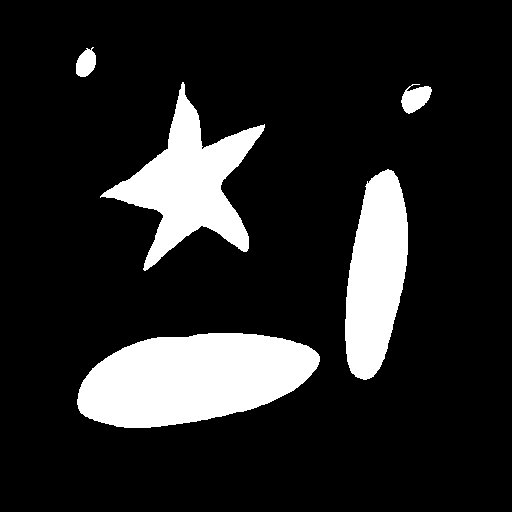

Supplement: S1 File — (ZIP) [file pcbi.1007758.s007.zip › SCRIPTs_and_GUIs/IMAGES for PCA GUI/manual10.png]

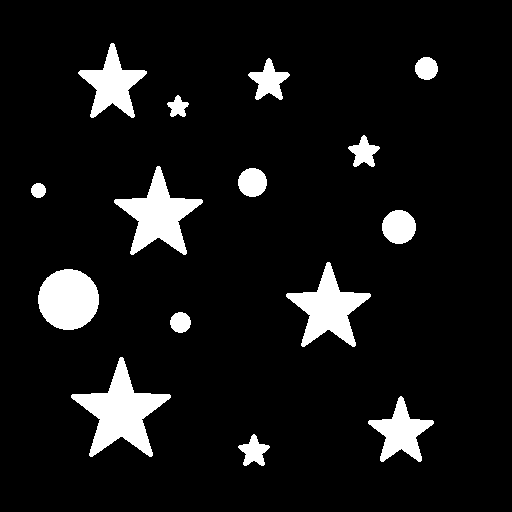

Supplement: S1 File — (ZIP) [file pcbi.1007758.s007.zip › SCRIPTs_and_GUIs/IMAGES for PCA GUI/stars9.png]

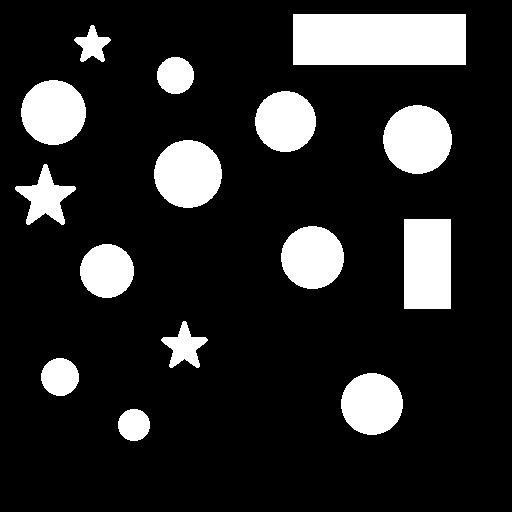

Supplement: S1 File — (ZIP) [file pcbi.1007758.s007.zip › SCRIPTs_and_GUIs/IMAGES for PCA GUI/misc14.png]

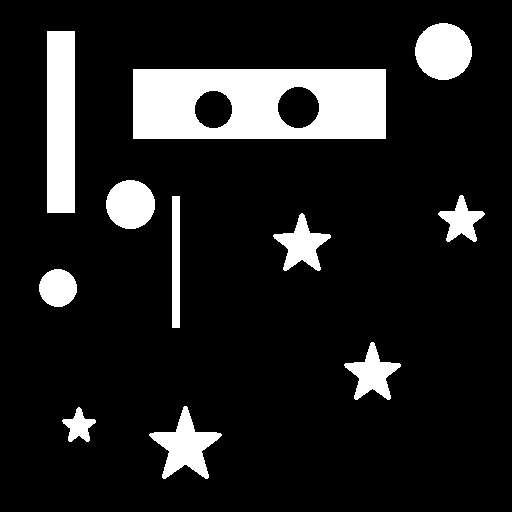

Supplement: S1 File — (ZIP) [file pcbi.1007758.s007.zip › SCRIPTs_and_GUIs/IMAGES for PCA GUI/misc15.png]

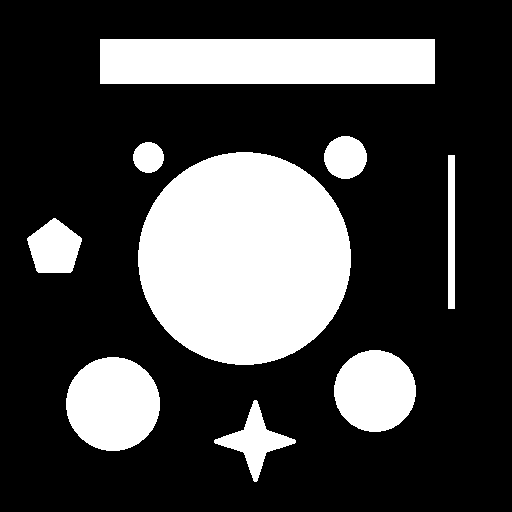

Supplement: S1 File — (ZIP) [file pcbi.1007758.s007.zip › SCRIPTs_and_GUIs/IMAGES for PCA GUI/misc17.png]

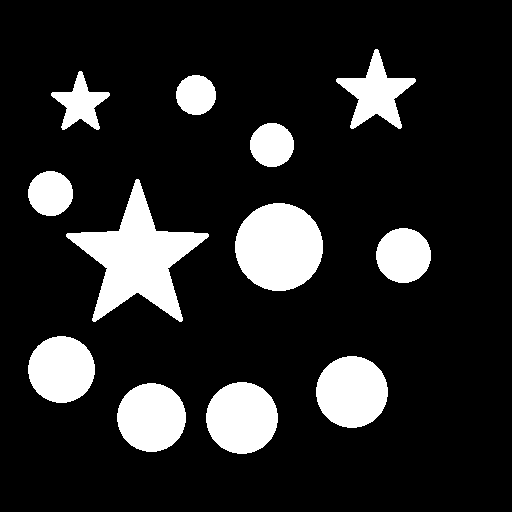

Supplement: S1 File — (ZIP) [file pcbi.1007758.s007.zip › SCRIPTs_and_GUIs/IMAGES for PCA GUI/misc16.png]

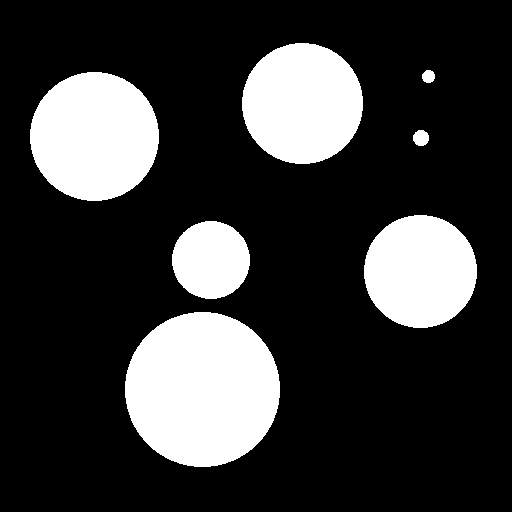

Supplement: S1 File — (ZIP) [file pcbi.1007758.s007.zip › SCRIPTs_and_GUIs/IMAGES for PCA GUI/circles2.png]

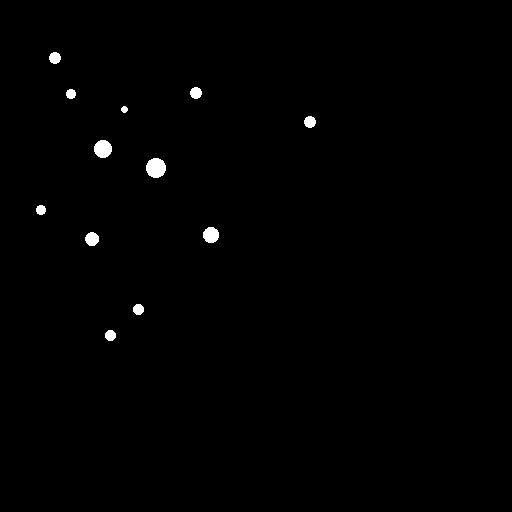

Supplement: S1 File — (ZIP) [file pcbi.1007758.s007.zip › SCRIPTs_and_GUIs/IMAGES for PCA GUI/circles3.png]

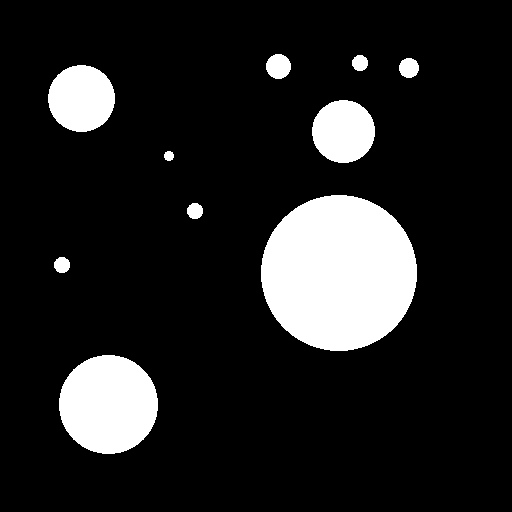

Supplement: S1 File — (ZIP) [file pcbi.1007758.s007.zip › SCRIPTs_and_GUIs/IMAGES for PCA GUI/circles1.png]

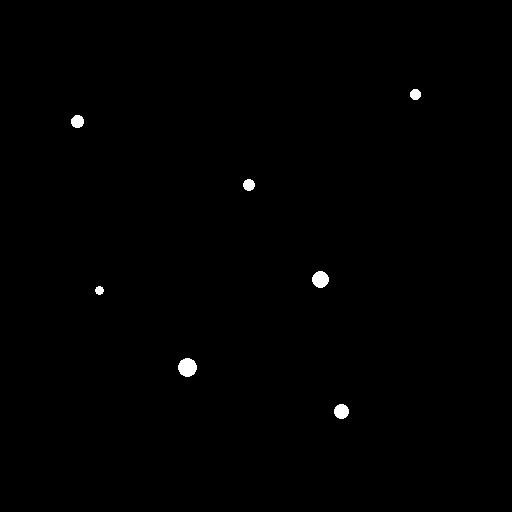

Supplement: S1 File — (ZIP) [file pcbi.1007758.s007.zip › SCRIPTs_and_GUIs/IMAGES for PCA GUI/circles4.png]

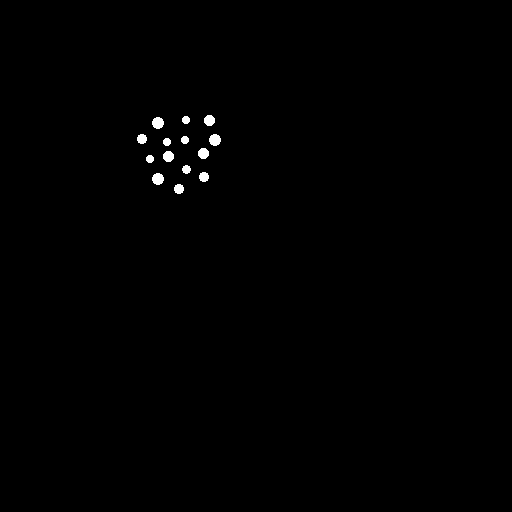

Supplement: S1 File — (ZIP) [file pcbi.1007758.s007.zip › SCRIPTs_and_GUIs/IMAGES for PCA GUI/circles5.png]

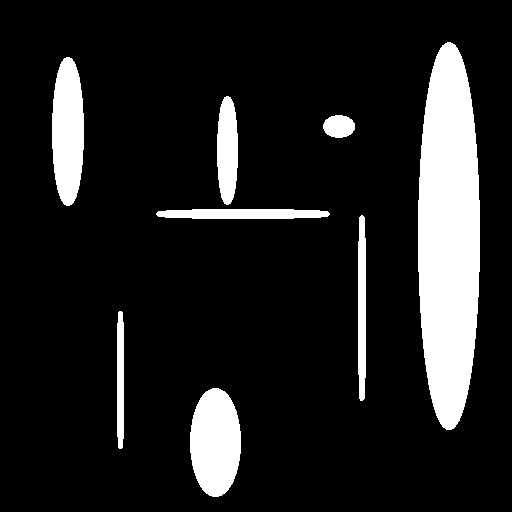

Supplement: S1 File — (ZIP) [file pcbi.1007758.s007.zip › SCRIPTs_and_GUIs/IMAGES for PCA GUI/ellipses8.png]

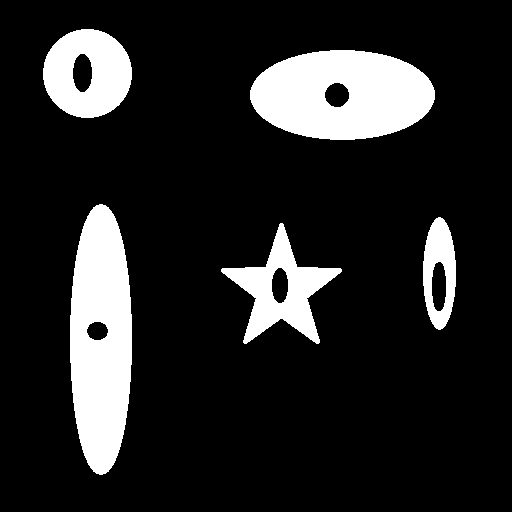

Supplement: S1 File — (ZIP) [file pcbi.1007758.s007.zip › SCRIPTs_and_GUIs/IMAGES for PCA GUI/hole12.png]

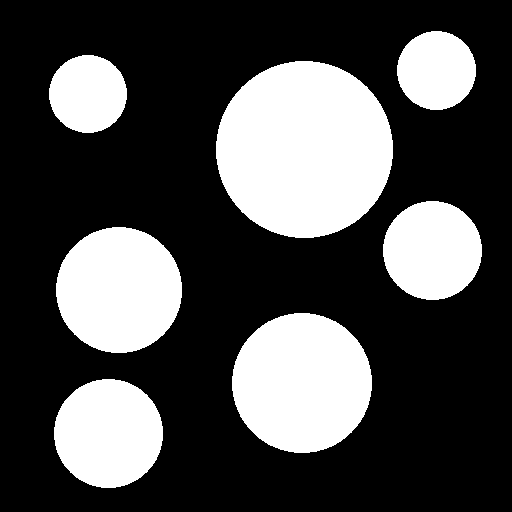

Supplement: S1 File — (ZIP) [file pcbi.1007758.s007.zip › SCRIPTs_and_GUIs/IMAGES for PCA GUI/circles6.png]

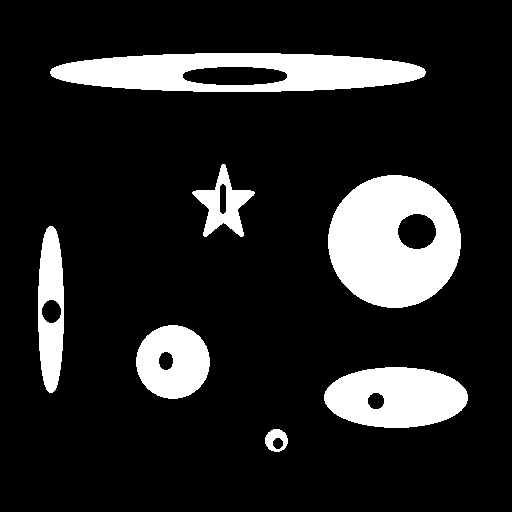

Supplement: S1 File — (ZIP) [file pcbi.1007758.s007.zip › SCRIPTs_and_GUIs/IMAGES for PCA GUI/hole13.png]
