# Supplementary material for: Shape-to-graph mapping method for efficient characterization and classification of complex geometries in biological images
Source: PLoS Comput Biol. 2020 Sep 3;16(9):e1007758. doi: 10.1371/journal.pcbi.1007758 (PMC7494120; doi:10.1371/journal.pcbi.1007758)
Supplement: S1 Table — http://cellprofiler-manual.s3.amazonaws.com/CellProfiler-3.0.0/modules/measurement.html. (DOCX) [file pcbi.1007758.s005.docx]

| Size and shape | | Measures area and shape features of identified objects |
| --- | --- | --- |
| 1 | Area | The number of pixels (2D) in the object |
| 2 | Compactness | The mean squared distance of the object's pixels from the centroid divided by the area |
| 3 | Eccentricity | The eccentricity of the ellipse that has the same second moments as the object |
| 4 | Euler number | The number of objects in the region minus the number of holes in those objects |
| 5 | Extent | The area of the object divided by the area of the object's bounding box |
| 6 | Form factor | $4\pi\cdot$Area/Perimeter ^2^ |
| 7 | Major axis length | The major axis of the ellipse with the same second central moments as the object |
| 8 | Maximum Feret diameter | The maximum possible distance between two parallel lines tangent on either side of the object |
| 9 | Maximum radius | The maximum distance of any pixel in the object to the closest pixel outside of the object |
| 10 | Mean radius | The mean distance of any pixel in the object to a pixel outside of the object |
| 11 | Median radius | The median distance of any pixel in the object to a pixel outside of the object |
| 12 | Minimum Feret diameter | The minimum possible distance between two parallel lines tangent on either side of the object |
| 13 | Minor axis length | The minor axis of the ellipse with the same second central moments as the object |
| 14 | Orientation | The angle in degrees between the x-axis and the major axis of the ellipse with the same second moments as the object |
| 15 | Perimeter | The total number of pixels (2D) around the boundary of each object in the image |
| 16 | Solidity | The proportion of the pixels in the convex hull that are also in the object |
| 17-46 | Zernike | Zernike shape features - the coefficients of the Zernike polynomials which describe a binary object. Includes polynomials from order 0 to 9. There are 30 Zernike shape features in total |
| Adjacent neighbors | | Measures for objects immediately adjacent to one another |
| 47 | Angle between neighbors | The angle formed between the centroids of the two adjacent objects with respect to the current object's center |
| 48 | First closest distance | The distance to the closest neighbor |
| 49 | Number of neighbors | The number of neighbors |
| 50 | Percent touching | Percent of the object's boundary which touch neighbors |
| 51 | Second closest distance | The distance to the second closest neighbor |
| Neighbors within 5 pixels | | Measures for objects adjacent after expansion by 5 pixels |
| 52 | Angle between neighbors | The angle formed between the centroids of the two adjacent objects with respect to the current object's center |
| 53 | First closest distance | The distance to the closest neighbor |
| 54 | Number of neighbors | The number of neighbors |
| 55 | Percent touching | Percent of the object's boundary which touch neighbors |
| 56 | Second closest distance | The distance to the second closest neighbor |
