# Supplementary material for: Shape-to-graph mapping method for efficient characterization and classification of complex geometries in biological images
Source: PLoS Comput Biol. 2020 Sep 3;16(9):e1007758. doi: 10.1371/journal.pcbi.1007758 (PMC7494120; doi:10.1371/journal.pcbi.1007758)
Supplement: S2 Table — These metrics are derived from the mean and standard deviation of the per-cell metrics described in Table 1, except for the dropped-off inner/outer boundary flags, giving the total of 4∙19 = 76 metrics. (DOCX) [file pcbi.1007758.s006.docx]

|  | **Measure Type** | **Feature Location** | **Feature Type** | **Feature** | **Description** |
| --- | --- | --- | --- | --- | --- |
| **1** | Mean | In-Graph | Width Profile | Mean | The mean radius along the root cycle |
| **2** | Mean | In-Graph | Width Profile | STD | Standard Deviation of the radius along the root cycle |
| **3** | Mean | In-Graph | Width Profile | Third Moment | Third Moment of the radius along the root cycle |
| **4** | Mean | In-Graph | Width Profile | Fourth Moment | Fourth Moment of the radius along the root cycle |
| **5** | Mean | In-Graph | Width Profile | Min | Minimum radius on the root cycle |
| **6** | Mean | In-Graph | Width Profile | Max | Maximum radius on the root cycle |
| **7** | Mean | In-Graph | Boundary Profile | Mean | Mean of the boundary profile measure across all points on the boundary |
| **8** | Mean | In-Graph | Boundary Profile | STD | Standard deviation of the boundary profile measure across all points |
| **9** | Mean | In-Graph | Boundary Profile | Max | Maximum boundary profile measure across all points on the boundary |
| **10** | Mean | In-Graph | Boundary Profile | AUC 0th Quartile | Area under the boundary profile curve |
| **11** | Mean | In-Graph | Boundary Profile | Number of Crossings 0th Quartile | Number of times the boundary profile reaches its minimum value. |
| **12** | Mean | In-Graph | Boundary Profile | AUC 25th Quartile | Area under the boundary profile curve above the 25th quantile |
| **13** | Mean | In-Graph | Boundary Profile | Number of Crossings 25th Quartile | Number of times the boundary profile goes above its 25th quantile. |
| **14** | Mean | In-Graph | Boundary Profile | AUC 50th Quartile | Area under the boundary profile curve above the 50th quantile |
| **15** | Mean | In-Graph | Boundary Profile | Number of Crossings 50th Quartile | Number of times the boundary profile goes above its 50th quantile. |
| **16** | Mean | In-Graph | Boundary Profile | AUC 75th Quartile | Area under the boundary profile curve above the 75th quantile |
| **17** | Mean | In-Graph | Boundary Profile | Number of Crossings 75th Quartile | Number of times the boundary profile goes above its 75th quantile. |
| **18** | Mean | In-Graph | General | Boundary Area | Area enclosed by the boundary |
| **19** | Mean | In-Graph | General | Boundary Perimeter | Perimeter of the boundary |
| **20** | Mean | Out-Graph | Width Profile | Mean | The mean radius along the root cycle |
| **21** | Mean | Out-Graph | Width Profile | STD | Standard Deviation of the radius along the root cycle |
| **22** | Mean | Out-Graph | Width Profile | Third Moment | Third Moment of the radius along the root cycle |
| **23** | Mean | Out-Graph | Width Profile | Fourth Moment | Fourth Moment of the radius along the root cycle |
| **24** | Mean | Out-Graph | Width Profile | Min | Minimum radius on the root cycle |
| **25** | Mean | Out-Graph | Width Profile | Max | Maximum radius on the root cycle |
| **26** | Mean | Out-Graph | Boundary Profile | Mean | Mean of the boundary profile measure across all points on the boundary |
| **27** | Mean | Out-Graph | Boundary Profile | STD | Standard deviation of the boundary profile measure across all points |
| **28** | Mean | Out-Graph | Boundary Profile | Max | Maximum boundary profile measure across all points on the boundary |
| **29** | Mean | Out-Graph | Boundary Profile | AUC 0th Quartile | Area under the boundary profile curve |
| **30** | Mean | Out-Graph | Boundary Profile | Number of Crossings 0th Quartile | Number of times the boundary profile reaches its minimum value. |
| **31** | Mean | Out-Graph | Boundary Profile | AUC 25th Quartile | Area under the boundary profile curve above the 25th quantile |
| **32** | Mean | Out-Graph | Boundary Profile | Number of Crossings 25th Quartile | Number of times the boundary profile goes above its 25th quantile. |
| **33** | Mean | Out-Graph | Boundary Profile | AUC 50th Quartile | Area under the boundary profile curve above the 50th quantile |
| **34** | Mean | Out-Graph | Boundary Profile | Number of Crossings 50th Quartile | Number of times the boundary profile goes above its 50th quantile. |
| **35** | Mean | Out-Graph | Boundary Profile | AUC 75th Quartile | Area under the boundary profile curve above the 75th quantile |
| **36** | Mean | Out-Graph | Boundary Profile | Number of Crossings 75th Quartile | Number of times the boundary profile goes above its 75th quantile. |
| **37** | Mean | Out-Graph | General | Boundary Area | Area enclosed by the boundary |
| **38** | Mean | Out-Graph | General | Boundary Perimeter | Perimeter of the boundary |
| **39** | STD | In-Graph | Width Profile | Mean | The mean radius along the root cycle |
| **40** | STD | In-Graph | Width Profile | STD | Standard Deviation of the radius along the root cycle |
| **41** | STD | In-Graph | Width Profile | Third Moment | Third Moment of the radius along the root cycle |
| **42** | STD | In-Graph | Width Profile | Fourth Moment | Fourth Moment of the radius along the root cycle |
| **43** | STD | In-Graph | Width Profile | Min | Minimum radius on the root cycle |
| **44** | STD | In-Graph | Width Profile | Max | Maximum radius on the root cycle |
| **45** | STD | In-Graph | Boundary Profile | Mean | Mean of the boundary profile measure across all points on the boundary |
| **46** | STD | In-Graph | Boundary Profile | STD | Standard deviation of the boundary profile measure across all points |
| **47** | STD | In-Graph | Boundary Profile | Max | Maximum boundary profile measure across all points on the boundary |
| **48** | STD | In-Graph | Boundary Profile | AUC 0th Quartile | Area under the boundary profile curve |
| **49** | STD | In-Graph | Boundary Profile | Number of Crossings 0th Quartile | Number of times the boundary profile reaches its minimum value. |
| **50** | STD | In-Graph | Boundary Profile | AUC 25th Quartile | Area under the boundary profile curve above the 25th quantile |
| **51** | STD | In-Graph | Boundary Profile | Number of Crossings 25th Quartile | Number of times the boundary profile goes above its 25th quantile. |
| **52** | STD | In-Graph | Boundary Profile | AUC 50th Quartile | Area under the boundary profile curve above the 50th quantile |
| **53** | STD | In-Graph | Boundary Profile | Number of Crossings 50th Quartile | Number of times the boundary profile goes above its 50th quantile. |
| **54** | STD | In-Graph | Boundary Profile | AUC 75th Quartile | Area under the boundary profile curve above the 75th quantile |
| **55** | STD | In-Graph | Boundary Profile | Number of Crossings 75th Quartile | Number of times the boundary profile goes above its 75th quantile. |
| **56** | STD | In-Graph | General | Boundary Area | Area enclosed by the boundary |
| **57** | STD | In-Graph | General | Boundary Perimeter | Perimeter of the boundary |
| **58** | STD | Out-Graph | Width Profile | Mean | The mean radius along the root cycle |
| **59** | STD | Out-Graph | Width Profile | STD | Standard Deviation of the radius along the root cycle |
| **60** | STD | Out-Graph | Width Profile | Third Moment | Third Moment of the radius along the root cycle |
| **61** | STD | Out-Graph | Width Profile | Fourth Moment | Fourth Moment of the radius along the root cycle |
| **62** | STD | Out-Graph | Width Profile | Min | Minimum radius on the root cycle |
| **63** | STD | Out-Graph | Width Profile | Max | Maximum radius on the root cycle |
| **64** | STD | Out-Graph | Boundary Profile | Mean | Mean of the boundary profile difference |
| **65** | STD | Out-Graph | Boundary Profile | STD | Standard deviation of the boundary profile difference |
| **66** | STD | Out-Graph | Boundary Profile | Max | Maximum of the boundary profile difference |
| **67** | STD | Out-Graph | Boundary Profile | AUC 0th Quartile | Area between the 0th quantile and the boundary difference curve |
| **68** | STD | Out-Graph | Boundary Profile | Number of Crossings 0th Quartile | Number of times the boundary difference curve crosses the 0th quantile |
| **69** | STD | Out-Graph | Boundary Profile | AUC 25th Quartile | Area between the 25th quantile and the boundary difference curve |
| **70** | STD | Out-Graph | Boundary Profile | Number of Crossings 25th Quartile | Number of times the boundary difference curve crosses the 25th quantile |
| **71** | STD | Out-Graph | Boundary Profile | AUC 50th Quartile | Area between the 50th quantile and the boundary difference curve |
| **72** | STD | Out-Graph | Boundary Profile | Number of Crossings 50th Quartile | Number of times the boundary difference curve crosses the 50th quantile |
| **73** | STD | Out-Graph | Boundary Profile | AUC 75th Quartile | Area between the 75th quantile and the boundary difference curve |
| **74** | STD | Out-Graph | Boundary Profile | Number of Crossings 75th Quartile | Number of times the boundary difference curve crosses the 75th quantile |
| **75** | STD | Out-Graph | General | Boundary Area | Area enclosed by the boundary |
| **76** | STD | Out-Graph | General | Boundary Perimeter | Perimeter of the boundary |
