# Supplementary material for: Shape-to-graph mapping method for efficient characterization and classification of complex geometries in biological images
Source: PLoS Comput Biol. 2020 Sep 3;16(9):e1007758. doi: 10.1371/journal.pcbi.1007758 (PMC7494120; doi:10.1371/journal.pcbi.1007758)
Supplement: S1 File — (ZIP) [file pcbi.1007758.s007.zip › SCRIPTs_and_GUIs/README.rtf]

This directory contains two GUIs (ShapeToGraph_GUI.m and PCA_GUI) that will let the user visualize and display various boundary metrics for a selected image and process multiple images for classification. There is an additional script (ExampleScript.m) which will show how one can programatically process an image and view the boundary profile for a selected region. There are also two functions (runVoronoi.m and analyzeGraph.m) that will allow one to easily construct the Graph, and process the boundaries for an image of their choosing. There will be additional details on running these scripts below. 

These scripts depend on code located in Common_Code, Voronoi_Analysis, and Voronoi_Construction, so please ensure that all three folders, and their subdirectories, are added to the path.

There are four subdirectories: 'Sample Images' contains examples of binary images that can be used to test the Voronoi Diagram code. 'Voronoi_Construction' contains code required to generate the diagram. 'Voronoi_Analysis' contains code to extract metrics from the boundaries. 'Common_Code' contains code that both stages depend on, including plotters and basic geometric calculations. These subdirectories will have a file explaining the primary functions involved.

IMPORTANT: Before running, the C++ code must be compiled into a file which MATLAB can run. A MATLAB script, BUILD_MEX_FILE.m, will perform this task for you. This may require installing additional software to compile the C++ code on your machine. Please see the "Supported Compilers" heading in BUILD_MEX_FILE.m for more information and further instructions. The build script will generate a Voronoi diagram for a test image after completion if successful.

==================================================================
1. USER INTERFACE

"ShapeToGraph_GUI.m" is an example user interface that allows one to quickly explore various metrics extracted from the Voronoi Diagram for a given image.

Running " ShapeToGraph_GUI.m " will launch an interactive figure window for displaying the Voronoi diagram. This window will contain three square axis windows at the top, two tables in the bottom left, and two rectangular axes in the bottom right. 

Select an image by pressing "import" at the top left corner of the figure window. This will open a file manager to let you select a binary image. Sample binary images are in the "Sample Images" folder (such as 'gtsmll2'). Once an image has been loaded, the Voronoi diagram will be generated, and all boundaries will be analyzed.

The three windows at the top of the GUI should now be populated. The top left window displays the binary image. The middle window shows the binary image with the full Voronoi diagram overlayed. This diagram is colored by subregions, sections of the Voronoi diagram associated with the nearest boundary. The right figure window will display the subregion for a selected boundary once a boundary has been selected. Initially, no boundary will be selected. UI controls to pan and zoom on these images are available in the top left of the window.

Dragging the slider will select different boundaries within the image. When a new boundary has been selected, the Voronoi diagram subgraph within the foreground and background will be displayed on the right figure window. The graph inside the object will be colored 'red', while the graph outside the object will be colored 'yellow'. The normalized boundary profile, which captures the shape of the boundary, and the radius along the root cycle, which captures the width of the object, will be plotted in the two axes below.

The table will contain two sets of twenty metrics for each boundary. Rows are different boundary IDs, and columns are different measurements. Most of these metrics are derived from the boundary profile and the radius distribution. The top table contains the boundary metrics extracted from the Voronoi Diagram inside the object (red in the right diagram), and the bottom contains the metrics extracted from the exterior Voronoi Diagram (yellow in the right diagram).
==================================================================
2.EXAMPLE SCRIPT

"ExampleScript.m" will display the Voronoi Diagram for a specific subregion in a pre-selected image, along with the boundary profile for this subregion. This script will demonstrate how to generate the Voronoi Diagram, and access raw metrics.

This script will load 'invbinary MAX intensity_DT2.tif' from the sample images folder, and then generate and analyze the Voronoi Diagram. 

It will then create a figure with two subplots. The left subplot contains a cropped region around a selected hole in the original image. The subregion associated with this hole will be drawn blue inside the object, and orange in the background. All other vertices and edges will be drawn grey. The right plot will draw the normalized boundary profile for this region. The green star in the boundary profile and in the diagram indicates the tip of the largest protrusion in this subregion.

The script will have comments indicating the explicit purpose of each block.
==================================================================
3.GRAPH CONSTRUCTION

runVoronoi.m is a function which will display the Voronoi diagram for a given binary image, and returns a 'records' structure which contains all the information about the graph. It has one optional numeric input, which will filter all holes in an image with an area below the provided value. 

When run, the user will be prompted to select an image. Example images are in the 'Sample Images' folder. The script will identify all boundaries in the image, and then construct the Voronoi diagram. The script will plot the Voronoi diagram in both the foreground and background, and output a large structure called 'records.' This is the primary input to other plotting functions, or graph analysis functions.

Further information about the records structure and the primary functions this stage involves are in the Voronoi_Analysis folder.
==================================================================
4.GRAPH ANALYSIS

analyzeGraph.m accepts the 'records' structure outputed by runVoronoi, and computes measures for all the boundaries. This outputs 'dataStruct', a large structure which contains very detailed information about all the boundaries, and 'statMat', a numBoundaries x 40 matrix containing 40 measurements extracted for each boundary.
