# Supplementary material for: Shape-to-graph mapping method for efficient characterization and classification of complex geometries in biological images
Source: PLoS Comput Biol. 2020 Sep 3;16(9):e1007758. doi: 10.1371/journal.pcbi.1007758 (PMC7494120; doi:10.1371/journal.pcbi.1007758)
Supplement: S1 File — (ZIP) [file pcbi.1007758.s007.zip › SCRIPTs_and_GUIs/Voronoi_Analysis/Information.rtf]

This directory contains the functions required to analyze the Voronoi Diagram. There are three primary functions which performs each stage of the analysis. These depend on helper functions located in the Processing folder.
These following functions are rain a sequence to extract all measures from the graph.
1.	processRecords
a.	This code accepts the records output, and identifies all edges and vertices which bisect different boundary IDs, referred to as bridges and hubs in the paper. See supplemental figure for more information. This returns a structure called dataStruct which contains boundary information from the graph.
2.	extractCycles
a.	This function accepts the record and dataStruct from processRecords. It loops through every subregion and performs a search to identify the root cycle, along with the path from all vertices to the root cycle. This is what computes the boundary profile and width distribution metrics. This updates and returns dataStruct.
i.	This calls extractSubGraphCycles, which is where the actual search is performed for an independent subregion.
ii.	The cycle info for each cycle can be retrieved with getCycleInfo(dataStruct, regionID).
1.	This returns two structures, cycleInner and cycleOuter. These correspond to processed graphs in the foreground and background.
3.	acyl_MajorAxis
a.	This function accepts the records and dataStruct from extractCycles, along with a third argument which is used to smooth the boundary profile in order to extract tips. This finds a central path for objects which have 'acyclic graphs,' corresponding to objects which lack holes inside them (or holes which lack objects inside them). This is purely to extract a more representative 'width distribution' metric for these type of objects. This updates and returns dataStruct.
4.	resampleAllInteriorCycles
a.	Accepts records and dataStruct from acyl_MajorAxis. This resamples the root cycle at evenly spaced intervals prior to extracting statistical measures. This updates dataStruct.
5.	dataStruct2Stats
a.	This accepts records and dataStruct, and computes 40 measurements for each boundary. This returns an N x 40 matrix, statMat, containing said features for each boundary. The specific features are in supplemental table 1.
The dataStruct Structure
dataStruct is a very large structure containing information about subregions in the graph. The most important information is the 'cycle' information extracted from extract cycles. 
The most important members in this structure will be detailed below, along with how to best access these members.
·	dataStruct
o	.loop – Contains analyzed subregion information
§	.object/.hole – Subregions located in the foreground or background
·	Each of these contain structures with information about this specific boundary. These are best extracted with getCycleInfo rather than through the structure directly. The contents for this are in the next list
·	.enclosed – Containing a list of all subregions which are enclosed by the boundary. (IE, subregion inside an object associated with the outermost boundary of said object)
·	.enclosing – Containing a list of all subregions which are 'enclosing' said boundary (IE, the subregion surrounding a hole inside an object) 
·	.acyclic – A subset of 'enclosed' subregions where the region has no interior cycles (IE, an isolated cell without any holes).
·	CycleInfo
o	This contains information about the boundary profile and width distribution for a given subregion.
o	Notation on vertexID
§	Global vertex ID – vertex ID with respect to the full Voronoi structure
§	Local vertex ID – vertex ID with respect to the local Voronoi structure
§	Boundary vertex IDs – vertex ID with respect to the position on the boundary.
§	Records2SubGraphMap_Vertices – maps all global IDs to local IDs
§	Subgraph2RecordsMap_Vertices – maps all local IDs to global IDs
§	boundaryVerts – maps all boundary vertex IDs to global IDs
o	.Vertices – Global VertexID for all nodes on the root cycle
o	.EL	- EdgeIDs for all edges on the root cycle
o	.radius – radius at each vertex in .Vertices
o	.isRootNode – binary vector indexed by global vertex ID. 1 if it is on the root cycle.
o	.parEdge – Indexed by local vertex ID. Gives the Edge ID which points towards the root
§	Each node on the cycle has a parent towards the previous node on the cycle. There are some boundaries in which the root cycle will contain the same vertex twice, reached via different edges. This is why this matrix is N x 2
o	.boundaryToCycleNode – Indexed by boundary vertex ID. Gives the global vertex ID for the ancestor node on the root cycle
o	.boundaryProfile – Distance from the root cycle to the boundary node, indexed by boundary vertex ID
o	.boundaryDiff – Distance from the root cycle to the boundary node, minus the radius at the ancestor root node. Indexed by boundary vertex ID.
o	.primaryMajAxis
§	Contains the radius, vertex list, edge list, and cumulative distance for the 'root cycle,' or 'root path' for objects which lack cycles.
o	.resampled
§	The position and radius at evenly sampled points on the root cycle.
