# Supplementary material for: Shape-to-graph mapping method for efficient characterization and classification of complex geometries in biological images
Source: PLoS Comput Biol. 2020 Sep 3;16(9):e1007758. doi: 10.1371/journal.pcbi.1007758 (PMC7494120; doi:10.1371/journal.pcbi.1007758)
Supplement: S1 File — (ZIP) [file pcbi.1007758.s007.zip › SCRIPTs_and_GUIs/Common_Code/Information.rtf]

This directory contains basic Geometric functions, along with plotting functions. This document will highlight the most commonly used plotting functions. 
Many functions accept a 'lineParams' argument. This is a cell array containing the arguments you would pass to line(…), such as 'LineThickness', or 'Color'
·	plotFromEdgeList
o	This is the core plotting function. It is used by the majority of other functions to actually draw the diagram.
o	Inputs:
§	EdgeListSubgraph - List of all edges to be included in the plot
§	Records – From Voronoi Construction
§	colorMat – Color to draw these edges
§	lineParams – See above
o	Outputs:	
§	Voronoi diagram drawn for the selected edges in the desired color, and handles to said plot
·	plotSubRegion
o	This plots the inner and outer boundaries for a specific subregion
o	Inputs
§	Records – from graph construction
§	regionID – number from 1 to records.numObjects indicating which subregion to plot
§	colorMat_Inner – color of the inner region (leave empty to skip plotting)
§	colorMat_Outer – color of the outer region (leave empty to skip plotting)
§	lineParams_Inner – Cell containing line parameters to pass to the plotting function (Empty cell or no argument to use defaults)
§	lineParams_Outer – Cell containing line parameters to pass to the plotting function for the outer subregion (Empty Cell or no argument to use defaults).
o	Outputs
§	Handle to the inner and outer plot functions
·	plotVoronoiSkeleton_SingleRegion
o	Inputs
§	Records – from graph construction
§	regionID – Object number ranging from 1 to records.numObjects. Regions are defined by the ID of the boundary enclosing them.
§	colorMat – Desired color of the diagram
§	lineParams – additional arguments for the plotter.
o	Outputs
§	Plots the Voronoi Diagram in the specified region and returns the plot handle
