# Supplementary material for: Shape-to-graph mapping method for efficient characterization and classification of complex geometries in biological images
Source: PLoS Comput Biol. 2020 Sep 3;16(9):e1007758. doi: 10.1371/journal.pcbi.1007758 (PMC7494120; doi:10.1371/journal.pcbi.1007758)
Supplement: S1 File — (ZIP) [file pcbi.1007758.s007.zip › SCRIPTs_and_GUIs/Voronoi_Construction/Information.rtf]

This directory contains the functions required to build the Voronoi Diagram. The majority of construction code is written in C++, and is located in the “MEX” folder. This code can be compiled to a MATLAB useable format, known as a “MEX” file. Information about compilation is in the “README” in the main folder.
Additional documentation about these files will be located in the file itself.
1.	imageVoronoi.m
a.	This code accepts a loaded binary image. This identifies all edges in the image between the foreground and background using multiLayerBorder, and then constructs the Voronoi Diagram for these edges using BuildVoronoi. This has a second argument, filtSize, which will fill all holes in the image smaller than this value. This returns the records structure, which contains all information about the edges and vertices in the graph. Information about the records structure will be at the bottom of this document.
2.	multiLayerBorder.m
a.	This accepts a binary image, and outputs a list of vertices and edges for all points along the boundary between the foreground and background, along with an 'imageStruct', which contains information about the boundaries, such as the boundary each vertex is uniquely associated with. All three output arguments are used as inputs to BuildVoronoi to construct the diagram. These points will be along 'half pixel boundaries,' rather than at the centroids of pixels at the edge.
b.	When two pixels are connected diagonally, there will be two vertices at the corner connecting them which overlap. To ensure that there are no overlapping vertices, and to ensure there are no zero-width regions, they are perturbed by a slight amount. The second argument to multiLayerBorder controls how much these points are perturbed. 1.0 indicates no perturbation (which will cause construction errors), 0.0 indicates maximum perturbation (in which the vertex will be set to the average position of it's neighboring points). See the supplemental figures for more information on this perturbation.
3.	BuildVoronoi.m
a.	This function accepts a list of vertices, edges, and the imageStruct extracted from multiLayerBorder. This function then calls VoronoiMEX, the compiled C++ code, and returns the records output. This function exists to simplify interacting with the VoronoiMEX function.
4.	testPerf_Def.m
a.	This is the test script which is run to validate that the code compiled correctly.
The Records Structure
Records is a very large structure containing all information about the graph. The following list will highlight the most important components of this structure.
·	records
o	.numObjects – Number of boundaries in the image
o	.imageMask – The binary image this diagram is associated with.
o	.vertexCoor – The input vertices
o	.EL – The input Edges.
o	.verts
§	A structure with multiple vectors for various features for each vertex. The index of each row is the VertexID for said vertex
§	.pos – (N x 2) x, y position
§	.rad – (N x 1) Distance to nearest input object 
§	.degree – (N x 1) Number of edges connected to said vertex.
§	.edgeIDList – (N x 4) The ID of the half edges connecting to said vertex. The maximum degree of a vertex is currently 4. If a vertex has fewer than four edges, then these columns will just be '0'. Most vertices will have three edges.
§	.color – (N x 3) Each vertex is the center of a circle tangent to three input elements. This gives the boundaryID associated with each element.
§	.length – (1 x 1) Number of Vertices
o	.edges
§	A structure which contains information about all the edges in the Voronoi Diagram. Each edge is represented by a half edge pair, where each half edge starts at opposing vertices and points in opposite directions. 
§	.twinID – (N x 1) The twin edge for each half edge
§	.origin – (N x 2) The vertex ID for this half edge and the twin half edge
§	.dist – (N x 1) The length of the edge.
§	.dir – (N x 2) The x and y direction of this edge at it's associated endpoint
·	Some edges, represented by .edgeType = 2, are actually parabolic arcs, and so the twin edge may not necessarily point in opposite directions
§	.edgeType – (N x 1) The type of edge
·	0 – Edge which connects to an input boundary vertex
·	1 – Straight edge
·	2 – Parabolic edge
§	.obj – (N x 1) The input objectID on the 'right' side of this edge
§	.color – (N x 1) The boundaryID for the associated objectID
§	.controlPoint – (N x 2) The x, y position for the control point associated with parabolic arcs, used primarily to draw the edge as a quadratic Bezier curve.
§	.length – (1 x 1) number of half edges
o	.inputs
§	(N x 5) matrix containing information about the input objects. This should not be required for most operations
§	Column 1: 1 if this is a vertex, 0 if it is an edge
§	Column 2: The boundaryID/color of the input
§	Column 3:
·	Vertex: X position
·	Edge: First endpoint ID
§	Column 4:
·	Vertex: Y position
·	Edge: Second endpoint ID
§	Column 5 – Associated FaceID (unused by this code)
o	.edgeSup
§	(N x 9) Matrix containing additional information about each input edge. This should not be required for most operations. 
§	Column 1: X direction
§	Column 2: Y direction
§	Column 3: Distance to the center of the sweep circle if said edge extended infinitely
§	Column 4,5,6: A, B, C if line is represented in general form
§	Column 7: The length of the edge
§	Column 8: Each edge is split into an 'upper' and 'lower' component, such that the right side of the edge points towards the center of the sweep circle. This is '1' if it is a lower edge, '0' if it is upper
§	Column 9: The twin edgeID for this edge.
